# Individual and Sociolinguistic Differences in Language Background Predict Stroop Performance

**DOI:** 10.3389/fcomm.2022.865965

**Published:** 2022-05-11

**Authors:** Max R. Freeman, Jonathan J. D. Robinson Anthony, Viorica Marian, Henrike K. Blumenfeld

**Affiliations:** 1 Language Acquisition and Bilingualism Lab, Department of Communication Sciences and Disorders, St. John’s College of Liberal Arts and Sciences, St. John’s University, Jamaica, NY, United States,; 2 Bilingualism and Cognition Laboratory, School of Speech, Language, and Hearing Sciences, College of Health and Human Services, San Diego State University, San Diego, CA, United States,; 3 Bilingualism and Psycholinguistics Research Group, Roxelyn and Richard Pepper Department of Communication Sciences and Disorders, School of Communications, Northwestern University, Evanston, IL, United States

**Keywords:** bilingualism, proficiency, age of acquisition, dominance, Stroop, cognitive control, inhibition, facilitation

## Abstract

To examine how differences in language experience and sociolinguistic context impact cognitive control, 146 Spanish-English bilingual participants were tested on a non-linguistic Stroop arrows task. Dimensions of language experience included a continuum of L2 proficiency, exposure, age of L2 acquisition, and English receptive vocabulary, along with cognitive non-verbal reasoning. Sociolinguistic context varied with more exposure to Spanish for participants in Southern California (SoCal) than in the Midwest. The task involved perceptual stimulus-stimulus conflict within stimulus features (e.g., right-pointing arrow on the left side of a display). Reaction times to trials where arrow location and direction matched (congruent), mismatched (incongruent), or arrow location was centered (neutral) were used to calculate Stroop (incongruent-congruent), facilitation (neutral-congruent), and inhibition (incongruent-neutral) effects. When examining performance on a continuum of bilingual language experience, individual differences in linguistic background (i.e., L2 proficiency and exposure, receptive vocabulary) and cognitive abilities (i.e., non-verbal reasoning abilities) predicted more efficient performance on the Stroop task. Across sociolinguistic contexts, findings revealed better performance via smaller Stroop and facilitation effects in the Midwest than in SoCal, and no group difference on the inhibition effect. We conclude that research on the cognitive consequences of bilingualism must consider a continuum of language experiences and must be situated in broader naturalistic contexts that take into account the sociolinguistic environments of language use.

## INTRODUCTION

Bilingual language experience may impact cognitive control (e.g., Luk et al., 2011; Lehtonen et al., 2018; Van den Noort et al., 2019; but see Paap et al., 2019; Beatty-Martínez et al., 2020). The debate on the cognitive consequences of bilingualism has been complicated by difficulties conceptualizing bilingualism due to variability in language background factors such as proficiency, exposure, sociolinguistic context of language use, and age of acquisition. One valuable approach to conceptualizing bilingualism has been a shift to assessing bilingualism on a continuum of these variables, instead of the bilingual-monolingual categorical distinctions (e.g., Luk and Bialystok, 2013; Kaushanskaya and Prior, 2015; Poarch and Krott, 2019; Kroll et al., 2021). The purpose of the current study was to examine whether individual differences and sociolinguistic context mediated cognitive control performance in individuals who varied in bilingual experience.

Cognitive control is the ability to regulate, plan, and execute goal-oriented behaviors (Braver, 2012) and involves the interplay between multiple executive functions (i.e., attention, cognitive flexibility, inhibitory control, working memory). Cognitive control is an important aspect of bilingual language processing (e.g., Green and Abutalebi, 2013), given that bilinguals navigate and manage two language systems that are active in parallel (e.g., Marian and Spivey, 2003; Starreveld et al., 2014). In fact, cognitive control correlates with bilinguals’ language use in the presence of conflicting crosslinguistic responses (e.g., Blumenfeld and Marian, 2013; Giezen et al., 2015; Singh and Mishra, 2016; Freeman et al., 2017) and is engaged during L2 processing (e.g., Darcy et al., 2015; Grant et al., 2015). Thus, bilingual experiences and contexts may promote cognitive control abilities due to the constant practice of monitoring and inhibiting language.

Van den Noort et al. (2019) cite 46 studies in the past 20 years that investigated bilingual vs. monolingual performance on tasks measuring cognitive control. The results demonstrate a bilingual advantage in 54% of studies, with 17% of studies revealing null effects. To account for differences in findings, research is beginning to examine how variability in bilingual experiences shapes cognitive control (e.g., Beatty-Martínez et al., 2020; Bonfieni et al., 2020). To accomplish this goal, multiple dimensions of bilingualism should be considered while targeting theoretically motivated aspects of cognitive control (e.g., Bonfieni et al., 2020). To examine contributions of proficiency, exposure, and age of acquisition to cognitive performance, variability along these dimensions can be leveraged within and across groups.

In the current study, we focused on how individual differences in language experience, proficiency, and cognition shaped cognitive control abilities in individuals with bilingual experience across sociolinguistic contexts. Specifically, we tested participants in the Midwest and in Southern California (SoCal) areas of the United States, thus varying the constellations of individual differences that contribute to each sociolinguistic context. Individual differences measures included linguistic variables, such as self-reported L2 proficiency and exposure, age of L2 acquisition, and receptive vocabulary, along with a cognitive measure of non-verbal intelligence, the Wechsler Abbreviated Scale of Intelligence (Wechsler, 1999). We used a non-linguistic Stroop arrows task that may be particularly sensitive to bilingual experience (Blumenfeld and Marian, 2014; Xia et al., 2021; but see Lehtonen et al., 2018; Paap et al., 2019).

The non-linguistic Stroop arrows task has been implemented across a number of studies to examine cognitive control abilities in bilinguals (e.g., Bialystok et al., 2008; Martin-Rhee and Bialystok, 2008; Bialystok and DePape, 2009; Blumenfeld and Marian, 2011, 2014; Giezen et al., 2015; Freeman et al., 2017). Participants identify arrow direction (left or right) when it appears on the left or right side on the visual display. Perceptual conflict results when the arrow direction and location do not correspond, such that a right-pointing arrow appears on the left side, or a left-pointing arrow appears on the right side of the visual display. Participants must resolve this perceptual conflict arising between the two dimensions of the stimulus on the display (location vs. direction of arrows) in order to respond appropriately. This type of conflict resolution has been termed stimulus-stimulus conflict, as the locus of the interference is within the stimulus (Kornblum et al., 1999). In addition, conflict occurs between stimulus dimensions and participant responses on Stroop-type tasks. For example, when a left-pointing arrow appears on the right side of a display, participants may initially be tempted to make a left-hand response, a response that must be inhibited together with the location dimension of the stimulus. Thus, Stroop tasks combine stimulus-stimulus and stimulus-response conflict (Kornblum et al., 1999).

Informed by the Dimensional Overlap Model (Kornblum et al., 1999), conflict between related stimulus dimensions (i.e., stimulus-stimulus: arrow direction left or right and arrow location left or right) is resolved at the same perceptual level as the related color-word Stroop task; in contrast, the traditional Simon task (Simon and Rudell, 1967) creates a conflict between a stimulus dimension and an unrelated manual response (i.e., stimulus-response: arrow direction up or down and button press left or right). Bilinguals have been shown to make more efficient responses on the Stroop than the Simon task, while monolinguals perform the same across the two tasks (Blumenfeld and Marian, 2014; Xia et al., 2021). Stroop arrows performance has also been found to correlate with bilingual language processing across a number of studies (Blumenfeld and Marian, 2011, 2013; Mercier et al., 2014; Giezen et al., 2015; Freeman et al., 2017). Specifically, the Stroop task with stimulus-stimulus conflict (e.g., inhibiting arrow location to identify arrow direction) may be more reflective of bilingual language experience (e.g., inhibiting one language while using the other).

Three related but separable Stroop processing effects were examined in the current study. The Stroop effect (i.e., inhibition and facilitation effects in combination, captured by incongruent minus congruent trials) has been found to correlate with speech and language processing in bilinguals (e.g., Blumenfeld and Marian, 2013; Giezen et al., 2015; Freeman et al., 2017). We maintain this overall effect in the current analyses, acknowledging that it may capture broader aspects of bilingual processing than its two subcomponents. Further, the Stroop facilitation effect was derived from response times on neutral minus congruent trials. This effect captures to what extent converging stimulus dimensions on congruent trials would facilitate responses relative to neutral trials. Here, neutral trials serve as a baseline where one of the stimulus dimensions is neutral (arrow location), meaning it never diverges from or converges with the other dimension (arrow direction). Finally, the Stroop inhibition effect was derived from responses on neutral minus incongruent trials. This effect captures to what extent conflicting stimulus dimensions on incongruent trials (arrow direction, arrow location) would trigger interference and the need to inhibit the arrow location dimension to respond correctly.

While some theoretical frameworks assign a shared mechanism to Stroop facilitation and inhibition and predict that the two effects track together (e.g., Botvinick et al., 2001), the two effects have been shown to be separable in studies where their timecourse was examined (e.g., Coderre et al., 2013; Parris, 2014). Critically, on a non-verbal Stroop arrows task, Hernández et al. (2010) found that Catalan-Spanish bilinguals showed larger Stroop facilitation but smaller Stroop inhibition effects relative to Spanish monolinguals. The authors took these findings as evidence that potential cognitive consequences of bilingualism may extend beyond inhibitory control to monitoring and making use of facilitatory information (also see Roelofs et al., 2006). In the current study, we examined performance on the Stroop arrows task across individuals with bilingual experience across two sociolinguistic contexts (Midwest and SoCal) to further specify how individual differences related to linguistic (i.e., proficiency, exposure, and age of L2 acquisition) and cognitive (non-verbal intelligence) factors shaped cognitive control abilities.

### Linguistic Background and Cognitive Control

Various dimensions of bilingualism have been observed to meaningfully characterize bilingual experience as it relates to cognitive control. First, *language proficiency* has been shown to mediate cognitive control abilities in adults. For example, Luque and Morgan-Short (2021) linked L2 proficiency with cognitive control performance for reactive inhibition and speed of processing on a letter-automatic continuous performance (AX-CPT) task, with higher L2 proficiency related to better inhibitory control. However, there was no relation between L2 proficiency and performance on an Eriksen flanker arrows task. In contrast, Xie (2018) found a relation between L2 proficiency and conflict monitoring on a flanker arrows task. Participants with higher L2 proficiency demonstrated faster reaction times than participants with lower L2 proficiency (for similar findings on language dominance metrics, see Goral et al., 2015; Robinson Anthony and Blumenfeld, 2019; but see Paap et al., 2019 for null findings). Based on these differences in findings examining the influence of L2 proficiency on cognitive control abilities, more research is necessary to characterize how L2 proficiency, along with other individual differences in participants’ language background and experience, shape cognitive control abilities.

One such additional individual differences variable that has been shown to influence cognitive control is L2 *age of acquisition* (AoA). Tao et al. (2011) found that Chinese-English bilingual adults outperformed their monolingual counterparts on a Simon-like (vertical arrows with a cueing component) task. Bilinguals with a late L2 AoA demonstrated greater conflict resolution skills, while early L2 AoA bilinguals demonstrated greater monitoring skills indexed by faster response times. Moreover, Luk et al. (2011) found that early bilinguals outperformed both later bilinguals and monolinguals on a flanker arrows task. It was anticipated in these studies that bilinguals would demonstrate better cognitive control; however, results suggested that a gradient of age of active bilingualism (derived from L2 AoA and age at testing) was a better predictor of cognitive control performance. Sabourin and Vinerte (2019) relatedly found that simultaneous French-English bilingual adults, but not early or late sequential bilingual adults, outperformed their monolingual peers on an arrow congruency task. Taken together, findings suggest that the timing and/or length of bilingual experiences (e.g., L2 AoA), as well as L2 proficiency, should be examined as predictors of cognitive control. The current investigation builds on previous findings to identify how *multiple* dimensions of bilingualism map onto specific aspects of cognitive control, while varying sociolinguistic contexts.

### Sociolinguistic Context and Cognitive Control

The adaptive control hypothesis posits that bilinguals respond adaptively to the demands on language use within their sociolinguistic environments (Green and Abutalebi, 2013). Indeed, a number of studies suggest that cognitive control is shaped by bilinguals’ contexts, such as whether they are exposed to primarily single language use vs. dual language use environments (Green and Wei, 2014; Green, 2018; Beatty-Martínez et al., 2020; Beatty-Martínez and Titone, 2021; Khodos and Moskovsky, 2021; Zhang et al., 2021). Here, we consider sociolinguistic context as a constellation of variables that constitute bilingual experience, such as L2 proficiency, exposure, and age of L2 acquisition. For example, in Beatty-Martínez et al. (2020) and Khodos and Moskovsky (2021), participants from different sociolinguistic contexts were distinguished from each other by their age of L2 acquisition and language exposure patterns.

Participants in Blumenfeld and Marian (2014) were tested on the non-linguistic Stroop arrows task and Simon task in the Midwest and SoCal sociolinguistic contexts. SoCal participants were in a sociolinguistic context where both languages were more regularly used, and they had learned Spanish earlier and reported higher Spanish proficiency than Midwest participants. SoCal residents live near the US-Mexican border, where many bilinguals are part of a binational bicultural context that frequently includes use of both languages (e.g., Cole, 2011). More balanced use of English and Spanish may have allowed SoCal participants to keep their two languages active simultaneously, while participants in the Midwest were exposed to Spanish less frequently. The observed distinction between the Midwest and SoCal contexts is also reflected in census data from the two communities. The SoCal area has a larger proportion of speakers of a language other than English (37.3%) and Spanish (21.4%), relative to the Midwest (language other than English: 17.2%; Spanish: 6.2%; census data extracted from mla.org language map). Sociolinguistic differences between the Midwest and SoCal participants in Blumenfeld and Marian likely contributed to the Midwest participants demonstrating better performance on the Stroop task relative to the Simon task, compared to smaller differences between Stroop and Simon performance in SoCal participants. SoCal participants were living in a context with a higher proportion of Spanish speakers who may have kept both languages available and relied less on stimulus-stimulus inhibition indexed by the Stroop task (i.e., non-target language inhibition). In the current study, we further examined the possibility that individual differences across dimensions of linguistic experience predicted inhibitory control performance within and across the same sociolinguistic contexts (the Midwest and SoCal).

### The Present Study

In the current investigation, we aimed to identify the individual differences in participants’ linguistic and cognitive backgrounds across sociolinguistic contexts (Midwest and SoCal) that led to more efficient performance on the non-linguistic Stroop arrows task (e.g., Blumenfeld and Marian, 2011, 2014; Giezen et al., 2015; Freeman et al., 2017). Individual differences information in participants’ language background and experience was obtained from the *Language Experience and Proficiency Questionnaire* (LEAP-Q, Marian et al., 2007), including linguistic factors of L2 proficiency and exposure and age of L2 acquisition (L2 AoA). For a more nuanced understanding, we considered participants on a bilingual continuum of L2 proficiency and exposure, as well as L2 AoA. We also administered the Peabody Picture Vocabulary Test-3 (Dunn and Dunn, 1997) to index receptive vocabulary in English (the L2 for all of our participants).

Beyond aspects of bilinguals’ linguistic environments, a cognitive measure of non-verbal intelligence was considered, the Wechsler Abbreviated Scale of Intelligence (Wechsler, 1999), since it has been shown to potentially contribute to performance in cognitive control. Evidence for a relation between non-verbal intelligence and interference resolution indexed by Stroop-like tasks has been mixed, with significant correlations identified in Chen et al. (2019) and Paap et al. (2020), but not in Mercier et al. (2014) or Paap et al. (2018). Non-verbal intelligence may therefore have the potential to contribute to variability whenever the influence of bilingual experience on cognitive control is examined. Non-verbal intelligence was included to better understand how it contributed to Stroop performance. Based on previous findings, we formulated the following predictions concerning Stroop performance and individual differences related to Stroop effects:

*First*, based on previous research using the Stroop arrows task (e.g., Bialystok et al., 2008; Martin-Rhee and Bialystok, 2008; Bialystok and DePape, 2009; Blumenfeld and Marian, 2011, 2014; Giezen et al., 2015; Freeman et al., 2017; Lehtonen et al., 2018; Xia et al., 2021), it was predicted that (a) all participants would show robust *Stroop effects* (i.e., be faster to respond to congruent than incongruent trials), *inhibition effects* (i.e., be faster to respond to neutral than incongruent trials), and *facilitation effects* (i.e., be faster to respond to congruent than neutral trials); and (b) when the dichotomous sociolinguistic context contrast was considered, no or small group differences would emerge across trial types (congruent, incongruent, and neutral).

*Second*, based on previous literature examining dimensions of bilingualism (e.g., Luk et al., 2011; Tao et al., 2011; Goral et al., 2015; Robinson Anthony and Blumenfeld, 2019; Luque and Morgan-Short, 2021) and cognitive abilities (e.g., Chen et al., 2019; Paap et al., 2020), we predicted that, *continuous* variables of L2 proficiency, exposure, acquisition age, receptive vocabulary, and non-verbal reasoning would shape performance on the non-linguistic Stroop arrows task. Specifically, we expected that (a) participants with greater self-reported L2 proficiency and exposure (composite variable), an earlier L2 AoA, and higher L2 proficiency (indexed by higher PPVT receptive vocabulary standard score) would demonstrate smaller Stroop, facilitation, and inhibition effects (e.g., Goral et al., 2015; Xie, 2018; Robinson Anthony and Blumenfeld, 2019; Luque and Morgan-Short, 2021).We also predicted that (b) participants with higher non-verbal reasoning abilities would demonstrate smaller Stroop, facilitation, and inhibition effects (e.g., Chen et al., 2019; Paap et al., 2020).

*Third*, based on previous findings that participants in SoCal are more proficient in and more frequently exposed to both languages than the Midwest participants (Blumenfeld and Marian, 2014), we predicted that differences across the two sociolinguistic contexts (Midwest and SoCal) in L2 proficiency and exposure and L2 acquisition would be linked to variation in performance on the Stroop task. Participants living in a sociolinguistic context in which both languages are regularly used, such as near the US-Mexican border in SoCal, may be less likely to rely on stimulus-stimulus inhibition on the Stroop task. Therefore, we expected that more efficient cognitive control would emerge in Midwest participants, where the two languages are relatively more separated, and that performance would be tied to bilingual experience.

## MATERIALS AND METHODS

### Participants

Participants included 146 Spanish-English heritage bilinguals. These participants were included from a larger sample of 235 bilinguals based on availability of self-reported language experience and proficiency variables in their L1 and L2 on the *Language Experience and Proficiency Questionnaire* (LEAP-Q) (Marian et al., 2007). Of the bilinguals, 64 were tested in the Midwest and 82 were tested in SoCal. All participants were native Spanish speakers who acquired English around the age of 5. Participants completed the LEAP-Q to capture self-reported L1 and L2 proficiency, current language exposure, and age of acquisition (AoA). Participants also performed the Peabody Picture Vocabulary Test-Third Edition (PPVT; Dunn and Dunn, 1997) to examine English receptive vocabulary skills, as well as the Wechsler Abbreviated Scale of Intelligence (WASI; Wechsler, 1999) to index non-verbal cognitive reasoning across participant sociolinguistic contexts and language groups, as well as. See Table 1 for linguistic and cognitive characteristics of the participant sample, de-aggregated by sociolinguistic context (Midwest and SoCal).

### Materials

#### Non-linguistic Stroop Arrows Task

**FIGURE 1 |:**
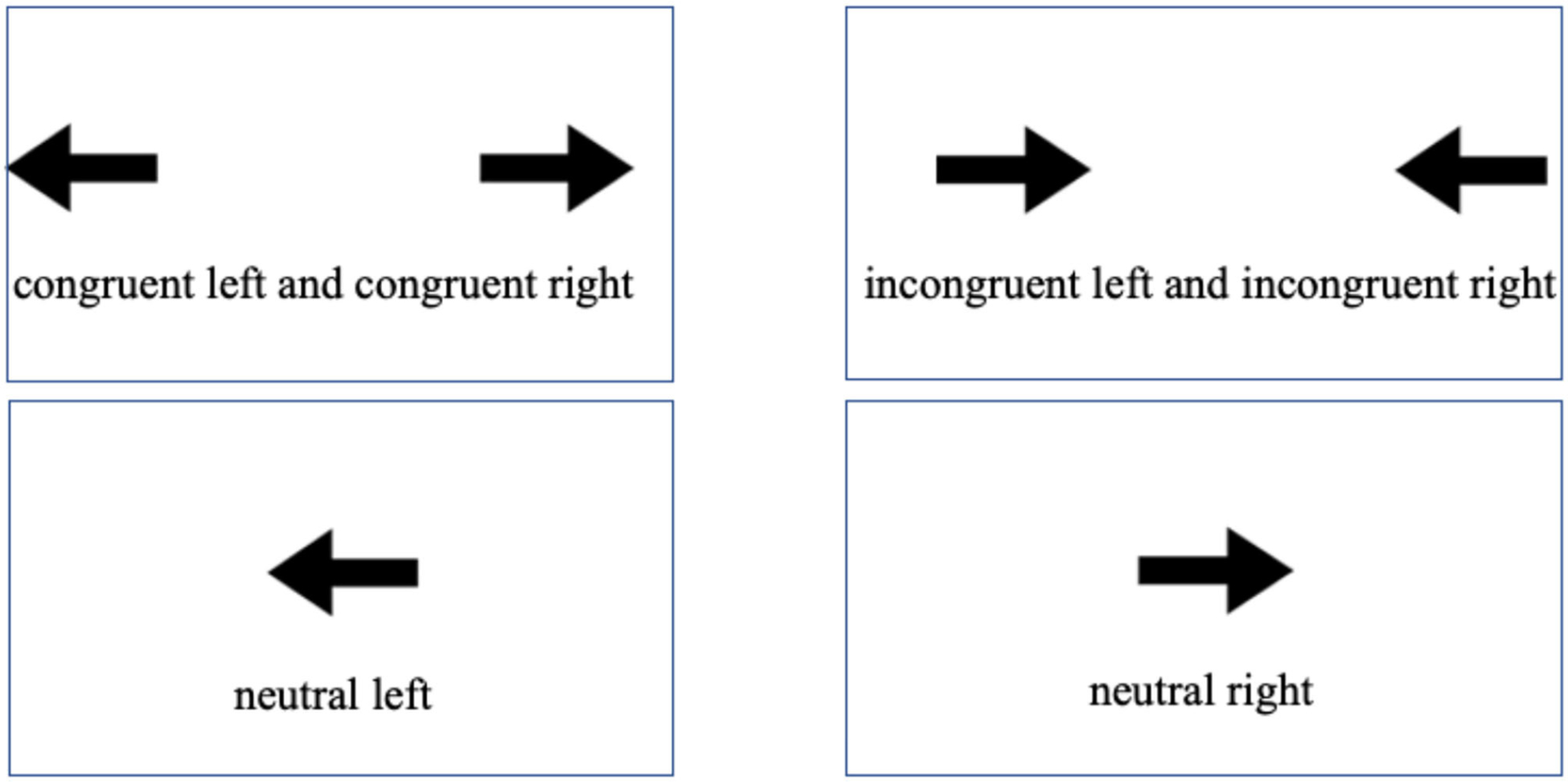
Congruent, incongruent, and neutral stimuli on the non-linguistic Stroop arrows task.

The non-linguistic Stroop task (e.g., Bialystok et al., 2008; Martin-Rhee and Bialystok, 2008; Bialystok and DePape, 2009; Blumenfeld and Marian, 2011, 2014; Giezen et al., 2015; Freeman et al., 2017) measured cognitive control abilities through the Stroop, facilitation, and inhibition effects. Congruent trials included arrows in which the location and direction corresponded. Incongruent trials contained arrows in which the location and direction did not correspond. Neutral trials comprised of arrows in the center of the visual display, pointing left or right (see Figure 1 for example congruent, incongruent, and neutral trials). The Stroop effect is defined as difference scores between incongruent and congruent trials, capturing the overall facilitation/inhibition effect. A smaller Stroop effect reflects participants’ ability to ignore irrelevant stimulus dimensions, thus incurring neither inhibition nor facilitation effects during the task. The facilitation effect is defined as difference scores between neutral and congruent trials, capturing participants’ ability to derive facilitative benefit from congruent stimulus dimensions. The inhibition effect is defined as difference scores between incongruent and neutral trials, capturing participants’ ability to resolve interference between incongruent stimulus dimensions.

In this retrospective study of the non-linguistic Stroop arrows task, three stimuls presentation platforms were employed in our labs at the time of testing across the Midwest and SoCal, including Matlab PsychToolbox (Brainard, 1997) (*n* = 64), ExperimentBuilder (SR Research Experiment Builder, 2011) (*n* = 60), and SuperLab (Cedrus Corporation, 2007) (*n* = 22). These stimulus presentation software packages are well-recognized platforms with excellent temporal resolution for chronometric data collection. The change of platform does not appear to have impacted the results; rather, the consistency across the platforms demonstrated the generalizability of the observed effects. The task consisted of 220 trials, including 20 practice trials (12 congruent, 4 incongruent, and 4 neutral). Black arrows were presented on a visual display pointing to the left, right, or center. The experimental trials contained 120 congruent, 40 incongruent, and 40 neutral trials. The congruent trials contained 60 trials with a leftward-facing arrow on the left side of the visual display and 60 with a right-ward facing arrow on the right side of the visual display. The incongruent trials contained 20 trials with a leftward-facing arrow on the right side of the visual display and 20 trials with a right-facing arrow on the left side of the visual display. The neutral trials contained 20 trials with a leftward-facing arrow in the center of the visual display and 20 trials with a rightward-facing arrow in the center of the visual display. The ratio of incongruent to congruent trials as well as neutral to congruent trials was 1:3.

### Procedure

All data were collected in a quiet room during in-person participation sessions in laboratory settings. Participants completed the non-linguistic Stroop arrows task (e.g., Blumenfeld and Marian, 2014; Giezen et al., 2015; Freeman et al., 2017), the Language Experience and Proficiency Questionnaire (LEAP-Q, Marian et al., 2007), the Wechsler Abbreviated Scale of Intelligence (WASI, Wechsler, 1999), and the Peabody Picture Vocabulary Test-Third Edition (PPVT, Dunn and Dunn, 1997).

On the non-linguistic Stroop arrows task, participants were instructed to ignore the location of the arrow on the visual display and respond on the keyboard using the left “Shift” key or right “Shift” key to indicate the direction of the arrow (left or right) as quickly and accurately as possible. For Matlab and Superlab versions of the script (*n* = 86), each trial began with a central fixation cross for 500 ms, followed by the stimulus display for 700 ms, and a blank screen for 800 ms. For the Experiment Builder script (*n* = 60), the blank screen was presented for 500 ms, followed by a 1,200 ms window where the stimulus was visible and responses could be made. As responses on the arrows Stroop task are typically made within the first 500 ms of stimulus presentation (e.g., Blumenfeld and Marian, 2014), and since response windows across the scripts were highly comparable at 1,500 and 1,200 ms, respectively, task version was not expected to influence performance. Trials were presented in a fixed pseudo-randomized order. Reaction times were measured from the onset of the stimulus display (arrow).

### Coding and Analysis

Reaction times and accuracy to congruent, incongruent, and neutral trials were analyzed. Reaction times below 200 ms and reaction times below or above 2.5 standard deviations from the mean were discarded. In addition, incorrect responses were not included within the reaction time analyses. Given that multiple sociolinguistic contexts were involved, and given our primary interest in examining Stroop, facilitation, and inhibition effects, we chose to adjust (i.e., standardize) reaction times to factor out any differences across participants in overall processing speed. Reaction times were first log-transformed (Baayen and Milin, 2010) and then standardized with sociolinguistic context (Midwest and SoCal) as a fixed factor. To standardize reaction times, we divided participants’ reaction times for each trial by their overall reaction time within that condition (congruent, incongruent, or neutral). To compare participants’ performance across congruent, incongruent, and neutral trials, linear mixed effects regression models were employed using the lme4 package in R (Bates et al., 2011). Error terms, random intercepts, and slopes included trial type and subjects.

We computed Stroop, facilitation, and inhibition effects based on log-transformed and standardized reaction times to congruent, incongruent, and neutral trials. Next, we conducted linear regression analyses to examine the influence of individual differences measures on Stroop, facilitation, and inhibition effects. Within our sample of 146 bilinguals, individual difference measures of interest included (1) sociolinguistic context (Midwest and SoCal), (2) self-reported L2 proficiency and exposure (a composite variable including L2 understanding, listening, and reading proficiency and current L2 exposure from the LEAP-Q), (3) age of L2 acquisition (AoA), (4) objective L2 proficiency, indexed by performance on the PPVT, and (5) WASI scores. Proficiency and exposure were combined given their robust correlation in the current sample (*r*^2^ = 0.80) as well as in previous research (Marian et al., 2007).

Forward stepwise linear regressions (Gareth et al., 2013) in R (R Core Team, 2020) were used to identify potential predictors of the Stroop, facilitation, and inhibition effects (individually) with the following candidate individual difference variables: L2 proficiency/exposure, L2 AoA, PPVT, and WASI. At each step, variables were chosen according to their contribution to the models’ R^2^ values. The stopping rule that limited the size of the final model was based off the lowest RMSE and MAE values, which are the prediction errors of each model. The lower the RMSE and MAE values, the better the model. For each effect, the best model contained one variable. Therefore, we examined the effect of the individual difference variables individually across Stroop, facilitation, and inhibition effects.

## RESULTS

Overall reaction times to congruent, incongruent, and neutral trials, as well as Stroop, facilitation, and inhibition effects, were reported in initial models with a categorical variable of context (Midwest and SoCal). This was followed by analyses to examine the modulating role of linguistic dimensions of bilingualism and cognitive individual differences measures on Stroop performance and to elucidate the source of sociolinguistic context (Midwest and SoCal) effects. The continuous individual differences measures included L2 proficiency/exposure, L2 AoA, L2 English receptive vocabulary (PPVT), and non-verbal reasoning (WASI). Raw accuracy rates to congruent, incongruent, and neutral trials were also reported.

### Overall Reaction Times in the Non-linguistic Stroop Arrows Task

A linear mixed effects regression model was used to analyze log-transformed standardized reaction times (RTs) to congruent, incongruent, and neutral trials with sociolinguistic context (Midwest and SoCal) as a fixed factor. Error terms, random intercepts, and slopes included trial type and subjects, see Table 2 for model statistics. Overall, RTs differed as expected across congruent, incongruent, and neutral trial conditions. Participants were fastest on congruent trials (log-transformed and standardized, *M* = 0.989, *SE* = 0.0002), slowest on incongruent trials (*M* = 1.017, *SE* = 0.0003), and slower on neutral (*M* = 0.997, *SE* = 0.0003) than congruent trials (all *p*s < 0.001, see Table 2).^1^

There were interactions between trial type and sociolinguistic context. Participants from SoCal (*M* = 0.988, *SE* = 0.0003) were marginally faster than participants from the Midwest (*M* = 0.989, *SE* = 0.0002) on congruent trials, B = −0.0008, SE = 0.0004, *t* = −1.943, *p* = 0.052. Yet for incongruent and neutral trials, participants from the Midwest were faster than participants from SoCal (incongruent trials: Midwest *M* = 1.015, *SE* = 0.0006; SoCal *M* = 1.018, *SE* = 0.0003; B = 0.0051, SE = 0.0007, *t* = 5.833, *p* < 0.001; neutral trials: Midwest *M* = 0.994, *SE* = 0.0006; SoCal *M* = 0.999, *SE* = 0.0004; B = 0.0040, SE = 0.0007, *t* = 6.898, *p* < 0.001).

### Influence of Sociolinguistic Context on Stroop, Facilitation, and Inhibition Effects

**FIGURE 2 |:**
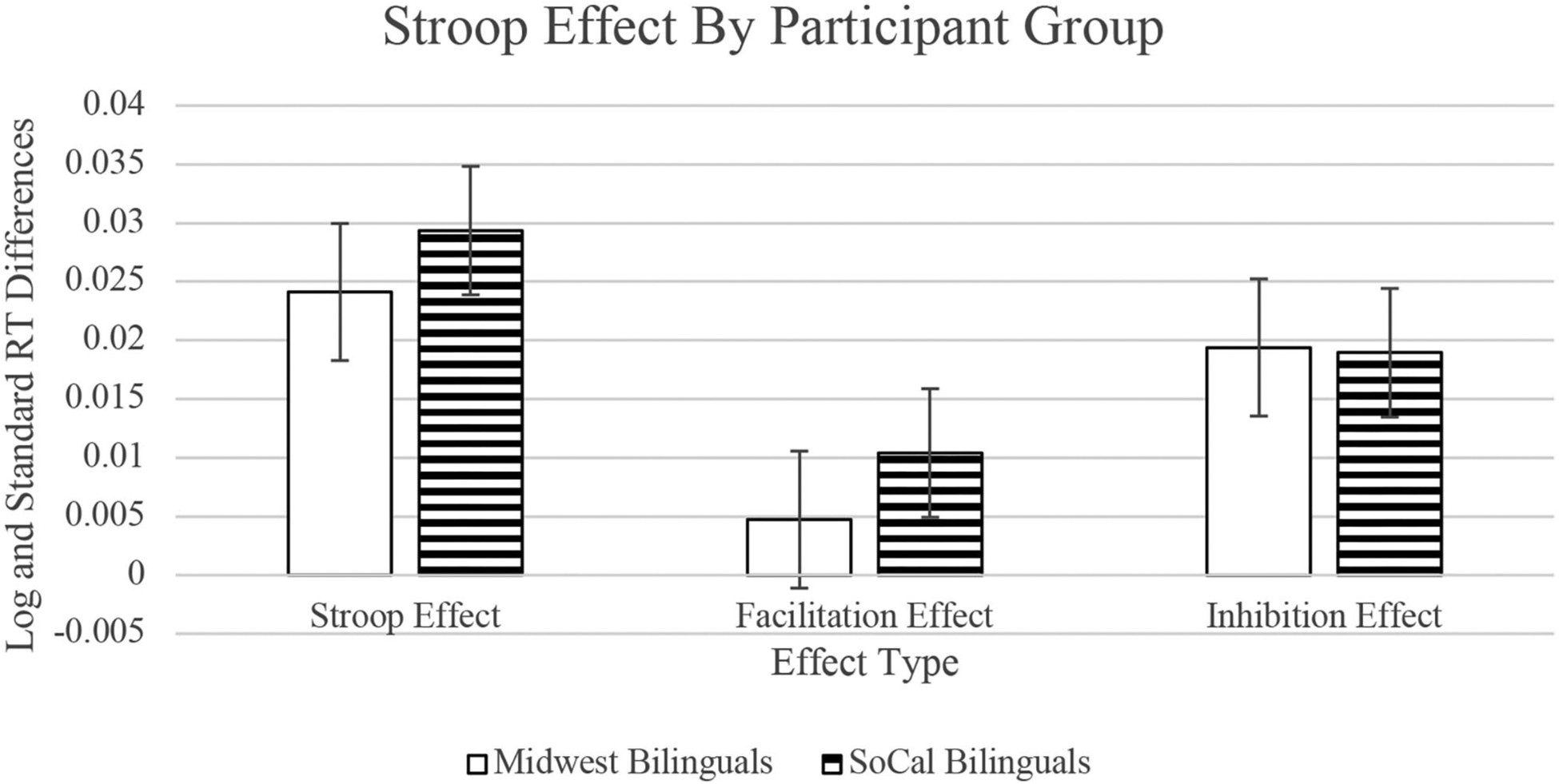
Stroop, facilitation, and inhibition effects across sociolinguistic contexts (Midwest and SoCal). Error bars represent 1 standard error. RT = reaction times.

For the **Stroop effect**, there was a main effect of sociolinguistic context, B = 0.0047, SE = 0.0020, *t* = 2.325, *p* = 0.021. Participants from the Midwest demonstrated a smaller Stroop effect than participants from SoCal (see Figure 2). For the **facilitation effect**, there was a main effect of sociolinguistic context, B = 0.0053, SE = 0.0014, *t* = 3.746, *p* < 0.001. Participants from the Midwest had a smaller facilitation effect than participants from SoCal. For the **inhibition effect**, there was no main effect of sociolinguistic context, B = −0.0005, SE = 0.0014, *t* = −0.333, *p* = 0.739.

To better understand how individual differences in language experience, proficiency, and cognition may have driven the observed sociolinguistic context differences, follow-up analyses on Stroop, facilitation, and inhibition effects included individual differences measures. See Table 1 for means and group differences across individual differences measures. Individual differences measures were entered as independent variables, and Stroop, facilitation, and inhibition effects (derived from congruent, incongruent, and neutral trials) were entered as dependent measures. See Table 4 for a summary of main effects and interactions for sociolinguistic context and individual differences.

### Influence of Individual Differences on the Stroop Effect

**FIGURE 3 |:**
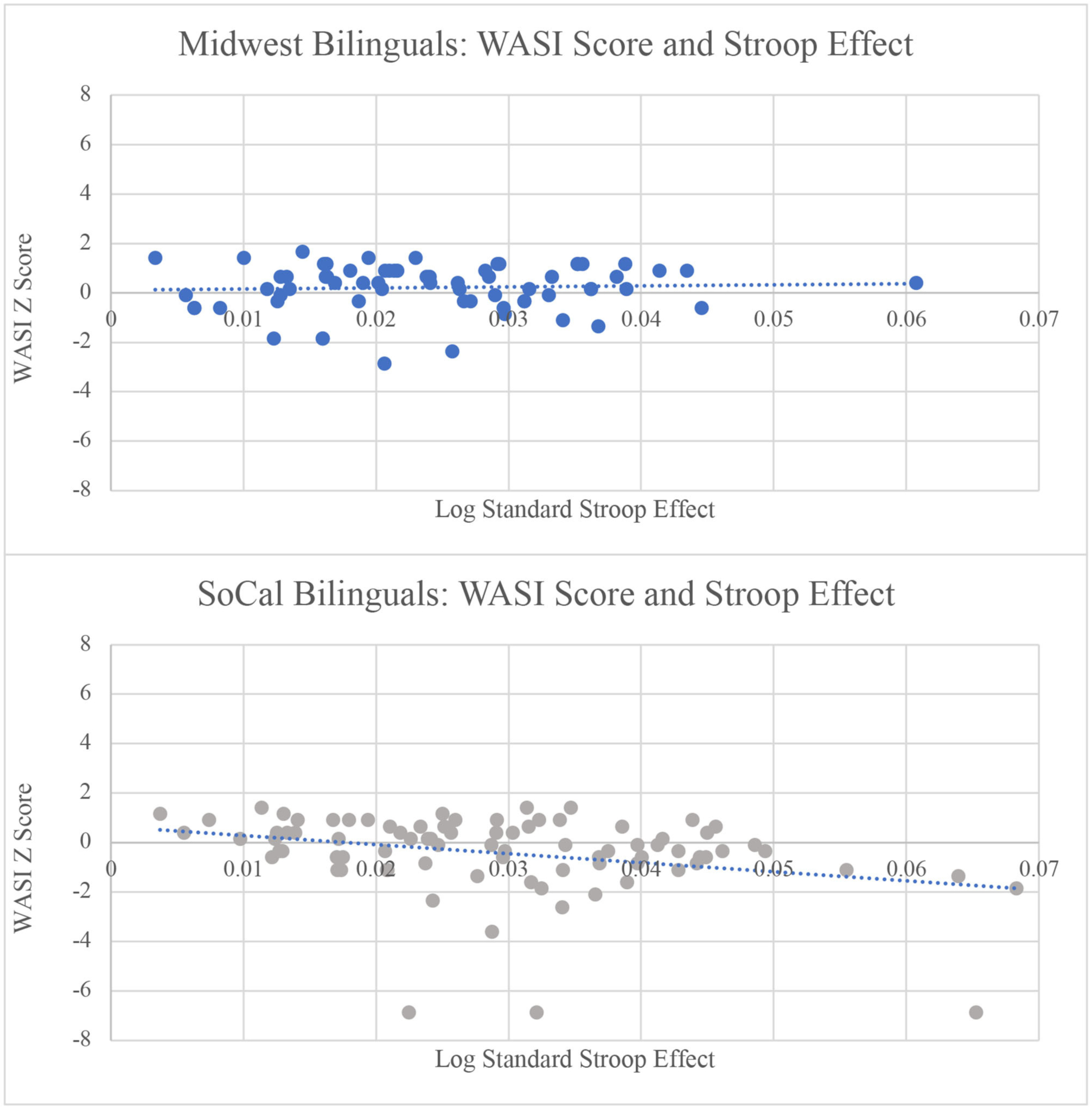
As WASI score increased, the Stroop effect decreased for SoCal bilinguals. This relation was not significant for Midwest bilinguals. RT = reaction time; WASI = Wechsler Abbreviated Scale of Intelligence matrix reasoning score.

Midwest bilinguals had higher L2 (English) proficiency and exposure than SoCal bilinguals (see Table 1). Accounting for L2 proficiency and exposure by entering the composite L2 proficiency/exposure variable into the **Stroop effect** model, a main effect of sociolinguistic context remained, B = 0.005, SE = 0.0021, *t* = 2.168, *p* = 0.032. Participants from the Midwest demonstrated a smaller Stroop effect than participants from SoCal, suggesting that the sociolinguistic context effect continued to be present when accounting for bilinguals’ proficiency and exposure. Moreover, bilinguals in the current sample had similar L2 AoAs across sociolinguistic contexts. When L2 AoA as a continuous measure was entered into the sociolinguistic context model, the previously-reported main effect of sociolinguistic context remained, B = 0.0081, SE = 0.0031, *t* = 2.601, *p* = 0.010, also suggesting that the location-based difference in the Stroop effect model did not change when considering L2 AoA. When examining PPVT performance as an index of L2 proficiency within the sociolinguistic context model for the Stroop effect, similar to proficiency/exposure and AoA, the main effect of sociolinguistic context survived, B = 0.0048, SE = 0.0020, *t* = 2.322, *p* = 0.022, suggesting that sociolinguistic context continued to be a predictor of the Stroop effect. Finally, when WASI scores were entered into the model, the main effect of sociolinguistic context became marginal, B = 0.0038, SE = 0.0001, *t* = 1.933, *p* = 0.055. There was also a main effect of WASI score, B = −0.0020, SE = 0.0009, *t* = −2.117, *p* = 0.031, suggesting that as WASI scores increased, the Stroop effect decreased. This pattern was led by SoCal bilinguals, as demonstrated by the interaction between WASI score and sociolinguistic context, B = −0.0046, SE = 0.0019, *t* = −2.412, *p* = 0.012. Therefore, higher non-verbal intelligence was associated with a smaller Stroop effect for SoCal bilinguals only, B = −0.0040, SE = 0.0013, *t* = −3.076, *p* = 0.003 (see Figure 3).

### Influence of Individual Differences on the Facilitation Effect

**FIGURE 4 |:**
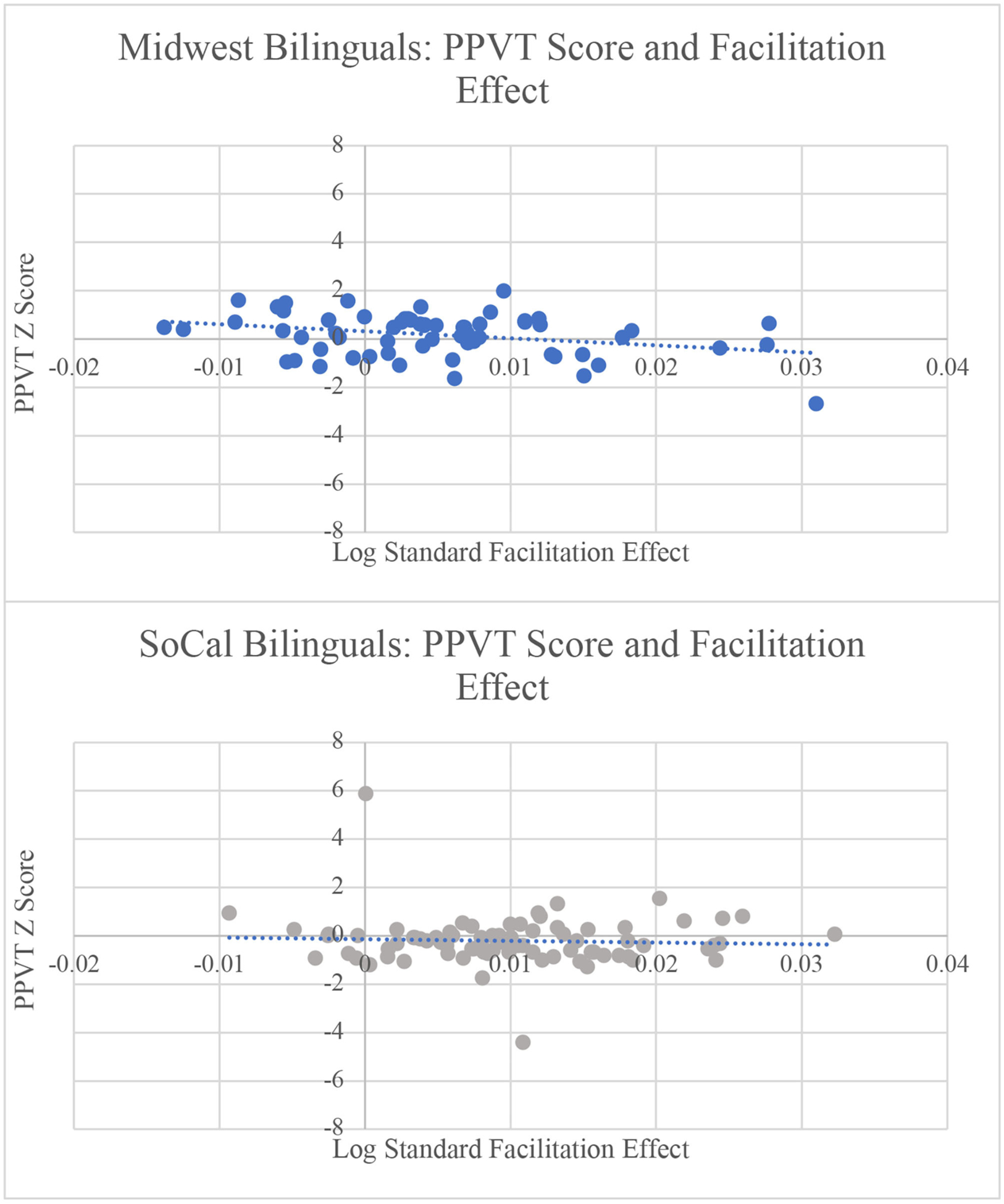
As PPVT/English receptive vocabulary score increased, the facilitation effect decreased for Midwest bilinguals. The relation was not significant for SoCal bilinguals. RT = reaction times; PPVT = Peabody Picture Vocabulary Test.

When examining L2 proficiency/exposure within the **facilitation effect** model, the main effect of sociolinguistic context remained, B = 0.045, SE = 0.001, *t* = 3.172, *p* = 0.002, in addition to a main effect of L2 proficiency/exposure, B = −0.001, SE = 0.0006, *t* = −2.590, *p* = 0.010. Participants in the Midwest had a smaller facilitation effect than participants in SoCal. As L2 proficiency/exposure increased, the facilitation effect decreased. For L2 AoA, the main effect of sociolinguistic context also was maintained, B = 0.0058, SE = 0.0021, *t* = 2.766, *p* = 0.006, demonstrating that L2 AoA did not drive differences in sociolinguistic context. For PPVT scores, there were main effects of sociolinguistic context, B = 0.0046, SE = 0.0014, *t* = 3.305, *p* = 0.001, PPVT score, B = −0.001, SE = 0.0007, *t* = −2.302, *p* = 0.023, and a marginal interaction of sociolinguistic context and PPVT score, B = 0.0029, SE = 0.0015, *t* = 1.949, *p* = 0.053. Sociolinguistic context continued to be a predictor of the facilitation effect. As PPVT scores increased, the facilitation effect decreased, a pattern led by the Midwest bilinguals, B = 0.0054, SE = 0.0013, *t* = 4.126, *p* < 0.001. Therefore, as receptive L2 vocabulary increased, the facilitation effect became smaller for Midwest bilinguals only (see Figure 4). Finally, for WASI scores, there were main effects of sociolinguistic context, B = 0.0048, SE = 0.0014, *t* = 3.402, *p* < 0.001, and WASI score, B = −0.001, SE = 0.0007, *t* = −2.060, *p* = 0.041. Sociolinguistic context continued to be a predictor of the facilitation effect when accounting for non-verbal intelligence. In addition, as WASI score increased, the facilitation effect decreased, suggesting that higher levels of non-verbal intelligence resulted in a smaller facilitation effect.

### Influence of Individual Differences on the Inhibition Effect

**FIGURE 5 |:**
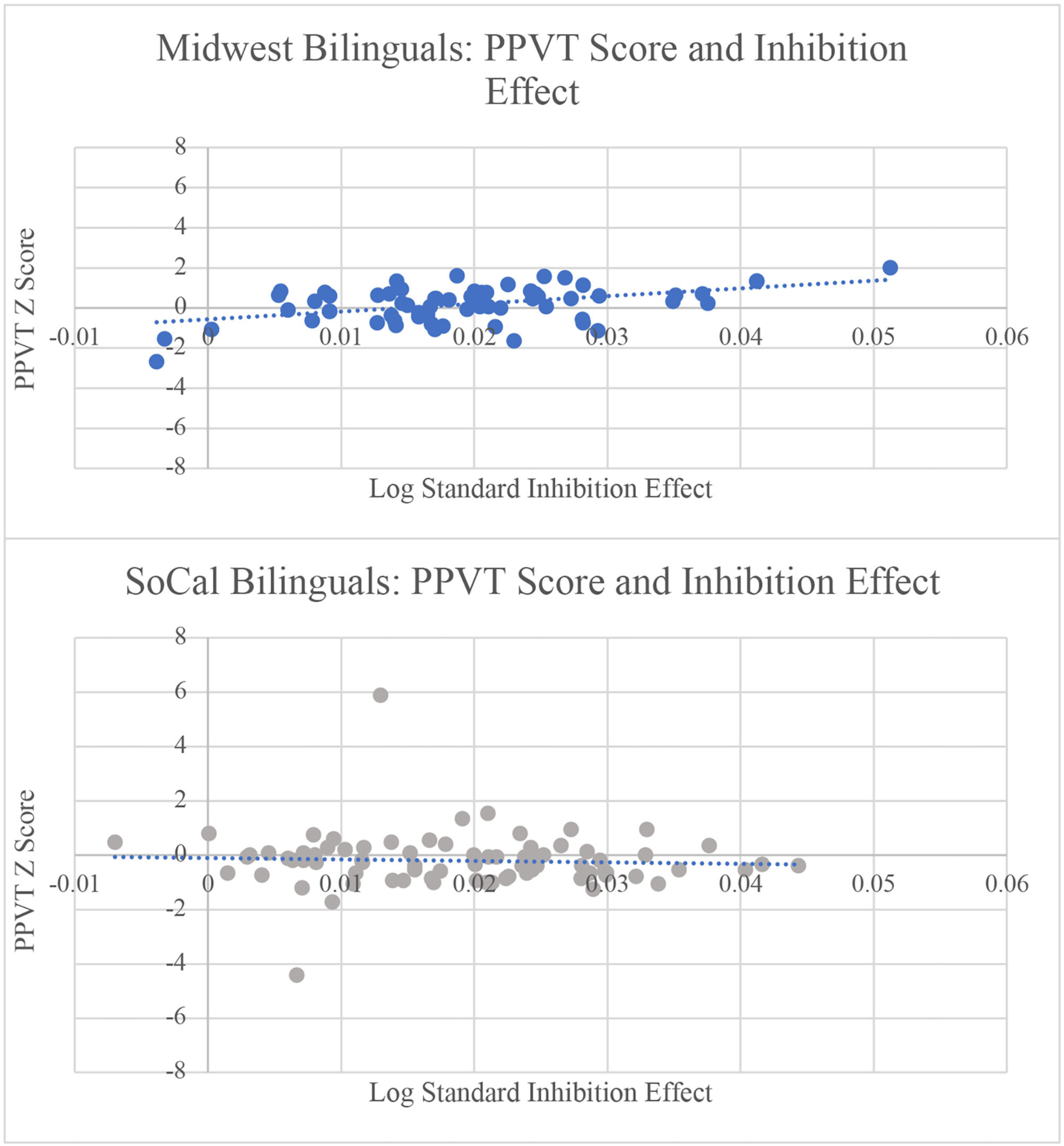
As PPVT score increased, the inhibition effect increased for Midwest bilinguals. This relation was not significant for SoCal bilinguals. RT = reaction times; PPVT = Peabody Picture Vocabulary Test.

**FIGURE 6 |:**
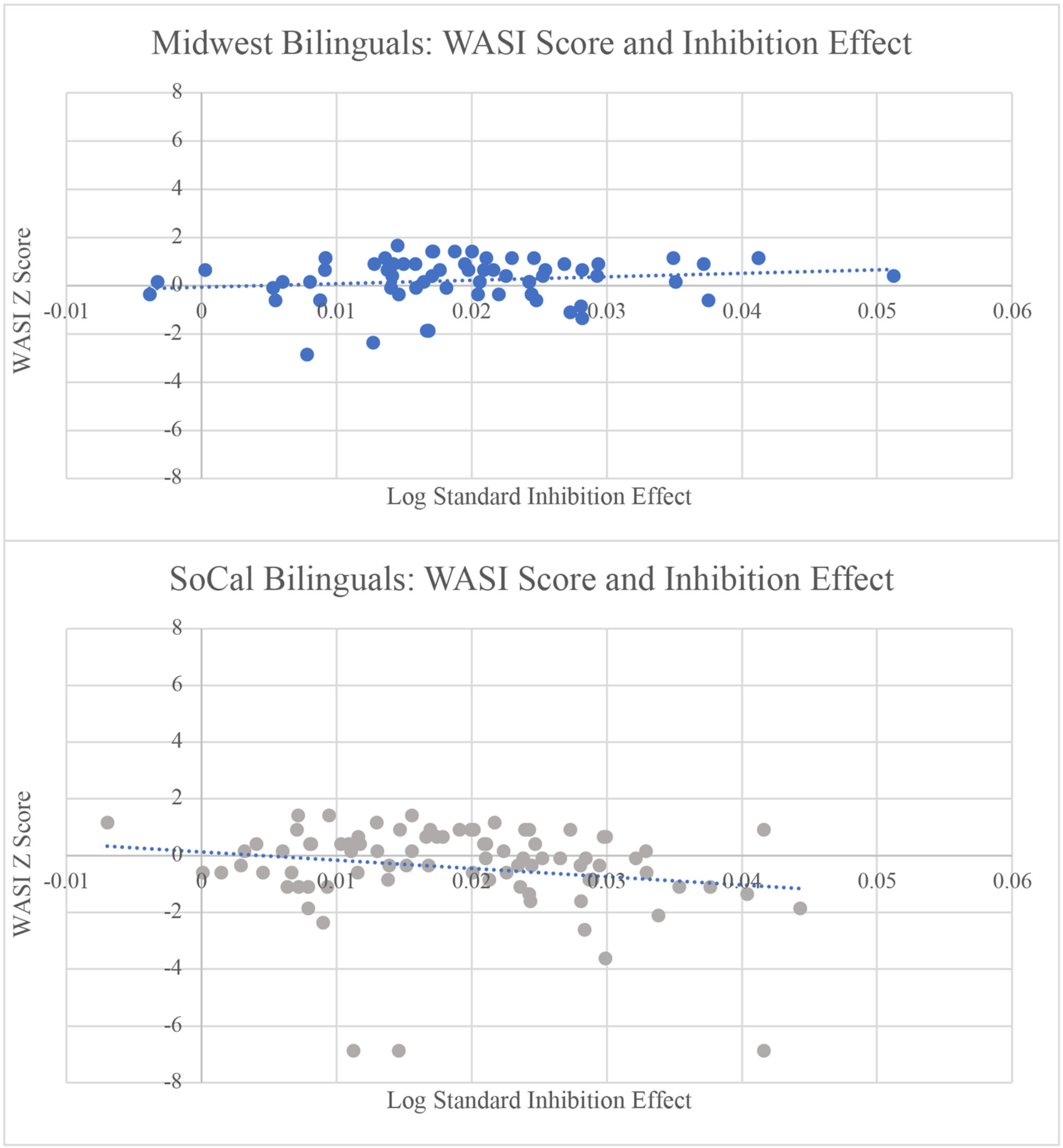
As WASI score increased, the inhibition effect decreased for SoCal bilinguals. This relation was not significant for Midwest bilinguals. RT = reaction times; WASI = Wechsler Abbreviated Scale of Intelligence matrix reasoning score.

When examining L2 proficiency/exposure as a predictor of the **inhibition effect**, the main effect of sociolinguistic context was insignificant, B = −0.0000, SE = 0.0010, *t* = −0.002, *p* = 0.998, as was the location effect for L2 AoA, B = 0.0022, SE = 0.0022, *t* = 0.838, *p* = 0.403; PPVT score, B = 0.0001, SE = 0.0017, *t* = 0.078, *p* = 0.9377; and WASI, B = −0.0009, SE = 0.0017, *t* = −0.558, *p* = 0.577. However, there was a main effect of PPVT score, B = 0.0020, SE = 0.0086, *t* = 2.405, *p* = 0.017, and an interaction of sociolinguistic context and PPVT score, B = −0.060, SE = 0.0017, *t* = −3.356, *p* = 0.001. As the PPVT score increased, the inhibition effect increased, a pattern led by the Midwest bilinguals B = 0.0054, SE = 0.0013, *t* = 4.126, *p* < 0.001. Therefore, for Midwest bilinguals, higher receptive vocabulary resulted in larger inhibition effects (see Figure 5). In addition, for WASI scores, an interaction emerged between WASI performance and sociolinguistic context, B = −0.0040, SE = 0.0016, *t* = −2.419, *p* = 0.017, suggesting that for SoCal bilinguals only, as WASI score increased, the inhibition effect decreased, B = −0.0024, SE = 0.0011, *t* = −2.225, *p* = 0.029. Thus, as non-verbal intelligence increased, the inhibition effect decreased for SoCal participants (see Figure 6).

### Overall Accuracy on the Non-linguistic Stroop Arrows Task

Finally, accuracy rates were analyzed across participants. A generalized linear mixed effects regression model was employed for log-transformed accuracy rates to congruent, incongruent, and neutral trials with the same fixed factors, error terms, random intercepts, and slopes as the reaction time model. The model failed to converge, and accuracy rates were overall high; we thus report means and standard errors across participant groups and conditions and limit analyses to reaction times (see Table 3).

## DISCUSSION

In the current study, we examined performance on the non-linguistic Stroop arrows task across a sample of Spanish-English bilinguals on a continuum L2 (English) experience in the Midwest and in Southern California (SoCal). Participants in these two groups were all Spanish heritage speakers immersed in English but differed in that the Midwestern participants reported greater exposure to their L2 (English) and were objectively more proficient in it, as reflected by PPVT scores. While Midwest bilinguals demonstrated smaller Stroop and facilitation effects than SoCal bilinguals, there was no difference in the inhibition effect across sociolinguistic contexts. Further, dimensions of bilingual experience, including self-reported L2 proficiency and exposure, receptive vocabulary, but not L2 AoA mediated Stroop performance, in addition to cognitive non-verbal reasoning. These mediating factors explain differences in location. The current results align with previous findings that cognitive control indexed by the Stroop task may be shaped by bilingual experience (Blumenfeld and Marian, 2014; Lehtonen et al., 2018; Xia et al., 2021, but see Paap et al., 2019) and cognitive factors (Chen et al., 2019; Paap et al., 2020).

The smaller Stroop effect in the Midwest participants was driven by a smaller facilitation effect compared to SoCal participants. This sociolinguistic context effect suggests that Midwest participants focused more exclusively on the relevant (arrow direction) instead of the irrelevant (arrow location) stimulus dimension that can facilitate or interfere with a correct response on the Stroop task. Further examining this overall effect, slightly faster responses on congruent trials and a larger facilitation effect were revealed in the SoCal participants. Midwest participants, on the other hand, demonstrated faster responses on neutral and incongruent trials. It thus appears that, at the group level, the Midwest and SoCal participants contrast in how they engaged cognitive control to perform on the Stroop task. The remaining Discussion further examines how individual difference factors (i.e., dimensions of bilingualism and cognitive abilities) and sociolinguistic context influence each of the Stroop, facilitation, and inhibition effects, as well as how sociolinguistic context may shape cognitive control more broadly.

### Influence of Individual Differences Measures on the Stroop Effect

We examined Stroop task performance with bilinguals on a continuum of L2 proficiency/exposure, L2 AoA, L2 proficiency via receptive vocabulary (PPVT scores), and non-verbal cognitive reasoning. In doing so, we considered whether and how continuous aspects of bilingualism and cognition could account for variability in Stroop performance. A smaller Stroop effect was observed in Midwest than SoCal participants. While none of the individual difference measures could fully account for the sociolinguistic context effect, it was modulated by WASI. For the SoCal participants only, as WASI score increased, the Stroop effect decreased, suggesting that better non-verbal cognitive reasoning abilities led to more efficient Stroop performance. This finding aligns with Chen et al. (2019) and Paap et al. (2020) who found a similar relation between Stroop performance and non-verbal cognitive reasoning. When looking across testing sites, Midwest participants had higher WASI scores than SoCal participants (*p* = 0.01), a difference likely accounted for by a combination of admission selectivity and university rankings at the two testing sites, as well as socioeconomic factors that may come into play in tuition rates across the universities (see Von Stumm and Plomin, 2015 for performance differences on cognitive tests across different socioeconomic samples). Therefore, within and between Midwest-SoCal settings, the Stroop effect was modulated by WASI.

### Influence of Individual Differences Measures on the Facilitation Effect

For the facilitation effect, increased L2 proficiency/exposure, higher PPVT scores, and better non-verbal reasoning were related to smaller facilitation effects. The facilitation effect decreased as L2 proficiency/exposure increased, a pattern that was found across locations and provides an explanation for the smaller facilitation effect in the Midwest context where L2 proficiency/exposure composite scores were significantly higher than in SoCal. In addition, L2 exposure was at 65% for Midwest participants and 55% for SoCal participants. PPVT scores could also account for the smaller facilitation effect in Midwest than SoCal bilinguals. For the Midwest participants only, the facilitation effect decreased as PPVT scores increased, aligning with previous findings that Stroop performance becomes more efficient with higher L2 proficiency (Xie, 2018; Luque and Morgan-Short, 2021). Indeed, we can note that the Midwest participants had higher PPVT scores (*M* = 109) than SoCal participants (*M* = 101) (see Table 2).

Moreover, increasing WASI scores were associated with a smaller facilitation effect, explaining the smaller facilitation effect in Midwest participants who also had somewhat higher WASI scores. Thus, greater L2 immersion, increased L2 proficiency, as well as increased cognitive skills are likely related to an increased ability to proactively monitor and attend to relevant information (e.g., bilinguals’ language in current use; the direction of the Stroop arrow in the current task) while ignoring irrelevant information (e.g., the language not currently in use; the facilitating arrow location on congruent trials).

Higher L2 proficiency/exposure, PPVT, and WASI scores were related to less reliance on the irrelevant stimulus dimension to facilitate responses to congruent (relative to neutral) trials. The current Stroop task consisted of 60% congruent, 20% incongruent, and 20% neutral trials, a ratio where it has been suggested that participants may engage a strategy that permits them to benefit from the facilitation provided by the irrelevant stimulus dimension on congruent trials (e.g., Gonthier et al., 2016). In theory, such a strategy would yield a greater facilitation effect. It is possible that individuals with higher L2 proficiency/exposure, PPVT, and WASI scores have more internal resources at their disposal because of greater cognitive capacity (Chen et al., 2019) and greater ease in maintaining the target language during L2 processing (Green, 1998). Therefore, individuals with these linguistic and cognitive profiles may focus on the relevant stimulus dimension on the Stroop task more consistently and thus rely less on facilitation cues during congruent trials. Relatedly, based on language profiles in the current study, Midwestern participants may operate more routinely in single-language contexts where information from the other (irrelevant) language must not be monitored. Individuals who routinely monitor input across two languages may also be more likely to monitor irrelevant information on non-linguistic tasks (Sabourin and Vinerte, 2019), resulting in larger facilitation effects for participants in SoCal. It is noteworthy that the current findings for SoCal bilinguals align with Hernández et al. (2010) in which Catalan-Spanish bilinguals demonstrated a larger facilitation effect relative to Spanish monolinguals. It is possible that the relatively integrated language contexts in Barcelona and SoCal may result in more continuous monitoring of information across languages and stimulus dimensions. Thus, linguistic factors (e.g., dimensions of bilingual experience; see Xie, 2018; Robinson Anthony and Blumenfeld, 2019; Luque and Morgan-Short, 2021) and cognitive factors (e.g., non-verbal intelligence; see Paap et al., 2018) may operate together to determine the facilitation effect.

### Influence of Individual Differences Measures on the Inhibition Effect

While there was no difference in the inhibition effect across locations, the inhibition effect was differentially modulated across sociolinguistic contexts by PPVT and WASI scores. Within Midwest participants, the inhibition effect became larger with increased PPVT scores. It is possible that the language profiles within the Midwestern context that tended toward greater L2 immersion and proficiency would rely less on inhibiting the non-target language, yielding larger inhibition effects in the non-linguistic domain. As a counterpoint to this pattern, Gullifer and Titone (2021) found that individuals in more linguistically diverse environments relied more on proactive control on an AX-CPT task (see Bice and Kroll, 2019, for similar findings). Based on these previous findings, participants in SoCal more successfully anticipate and inhibit incongruent information. This was found to be the case primarily in individuals with higher non-verbal reasoning scores, suggesting a cognitive component in the inhibitory control performance of the SoCal participants. In general, SoCal participants, relative to Midwest participants, demonstrated faster responses on congruent trials and overall slower responses on neutral and incongruent trials. SoCal participants may thus have engaged a monitoring strategy to respond to neutral and incongruent trials and to adaptively inhibit irrelevant stimulus dimensions.

The combined results across the Stroop, facilitation, and inhibition effects suggest that a constellation of linguistic and cognitive individual differences could explain the nature of sociolinguistic context contrasts. Therefore, examining the full sociolinguistic contexts where linguistic and cognitive factors co-exist can provide a more accurate understanding of how linguistic and cognitive individual differences operate together to determine performance.

### Influence of Sociolinguistic Context on Cognitive Control

Here, we sought to examine how participant differences across sociolinguistic contexts were shaped by the above-identified dimensions. In general terms, the influence of sociolinguistic context in our current study may be explained within the framework of the adaptive control hypothesis (Green and Abutalebi, 2013). Sociolinguistic contexts with varying demands on language interaction, such as contexts of competitive vs. cooperative language use (Beatty-Martínez and Titone, 2021), shape cognitive control performance in bilinguals. Given the language background information collected from participants in the current study, it is likely that the participants in SoCal functioned in a more integrated (cooperative) and linguistically diverse language context where both languages were used frequently, while the environment for participants in the Midwest reflected a potentially less integrated and potentially more competitive language environment in which the majority language was used more. The contrast between cooperative and competitive language environments made in Beatty-Martínez et al. (2020) focused on L2 immersion in the US (a competitive and varied context) vs. L1 immersion in Puerto Rico (a cooperative and integrated context). In the current investigation, we further home in on variability of L2 immersion contexts within the US. The Midwest bilinguals lived in a relatively more competitive (separated) language context than the SoCal bilinguals, who lived in a relatively more cooperative (integrated) language context.

Similar to the current study, the SoCal sample in Blumenfeld and Marian (2014) reported higher Spanish exposure than the Midwestern participants. Across Midwest and SoCal contexts, Blumenfeld and Marian compared performance on the Stroop arrows task to performance on a Simon task that did not include stimulus-stimulus inhibition (i.e., the need to inhibit an overlapping stimulus dimension of *right-left* location while making a *right-left* arrow direction judgment on the Stroop task). Better performance on the Stroop task, compared to the Simon task, emerged more robustly in participants who had been tested in the Midwest than those tested in SoCal. As in the current study, it is possible that the SoCal bilinguals were more likely to be in a cooperative L2 language immersion context in which their two languages were active simultaneously than participants in the Midwest. Therefore, SoCal participants may have relied less on linguistic stimulus-stimulus inhibition (e.g., inhibiting Spanish when using English) and found less opportunity to practice this suppression mechanism in their daily language use. The current findings align with recent work identifying distinctions in cognitive control between bilingual contexts where languages are used competitively (i.e., mostly separately) vs. cooperatively (Green and Wei, 2014; Green, 2018; Beatty-Martínez et al., 2020; Khodos and Moskovsky, 2021).

Investigations of the adaptive control hypothesis do not all converge on sociolinguistic contexts supporting cognitive control. Kałamała et al. (2020) found no relation between the degree of dual language context (e.g., believed to positively influence cognitive control) and inhibitory control (including latent variable and single measure outcomes on color/word Stroop, antisaccade, go/no go, and stop signal tasks; see Paap et al., 2019 for similar null findings). We take these studies to suggest that other yet unspecified factors likely contribute to shaping performance across bilingual populations. Testing for and establishing contrasts across multiple bilingual populations provides a valuable methodological avenue where the confluence of linguistic (dimensions of bilingualism) and cognitive (e.g., non-verbal intelligence) factors can be examined together to determine cognitive control performance. Future research is needed to describe how sociolinguistic contexts (e.g., extent of L2 immersion, Zhang et al., 2021) impact cognitive control (e.g., Gullifer et al., 2018; Ooi et al., 2018; Pot et al., 2018; Gullifer and Titone, 2020).

### Limitations and Future Directions

As others have suggested (e.g., Poarch and van Hell, 2019; Beatty-Martínez et al., 2020; Bonfieni et al., 2020), the cognitive control tasks employed when cognitive consequences of bilingualism are examined must incorporate the types of conflict (e.g., stimulus-stimulus inhibition) that reflect bilingual language experiences (e.g., inhibiting one language to use the other). Admittedly, the Stroop arrows task is a single measure of a very specific type of cognitive control. If it is indeed the stimulus-stimulus conflict nature of the Stroop task that simulates bilingual experience (Blumenfeld and Marian, 2014; Xia et al., 2021), then other tasks with these features might be employed to trace the dynamics of stimulus-stimulus inhibition from the linguistic into the non-linguistic domain. Further, future studies may include important sociolinguistic variables such as more nuanced metrics of long-term L2 immersion and cooperative vs. competitive use of language, social networks, and language attitudes. Considering the social and sociolinguistic aspects of bilingualism together with cognitive consequences is in its relative infancy as an approach (e.g., Guerrero and Luk, 2021). Yet, for a more accurate and comprehensive understanding of the cognitive consequences of bilingualism, a concurrence of linguistic and cognitive factors on a continuum of bilingualism (i.e., beyond categorical distinctions) must be examined.

## CONCLUSIONS

Here, we examined how cognitive performance on the non-linguistic Stroop arrows task was shaped by linguistic and cognitive factors across participants with various language experiences living in two sociolinguistic contexts. The differences in overall Stroop and facilitation effects, as well as the modulation of inhibition effects across the two sociolinguistic contexts, were driven by individual differences across a set of linguistic variables that together formed a multidimensional continuum of bilingualism. Specifically, the sociolinguistic context distinctions in Stroop performance could be explained by a constellation of individual differences in L2 experience and cognitive abilities. The individual differences that modulated Stroop, facilitation, and inhibition effects within and across language contexts included higher L2 proficiency and exposure (associated with smaller facilitation effects), higher L2 PPVT scores (associated with smaller facilitation and larger inhibition effects), as well as higher nonverbal reasoning scores (associated with smaller Stroop, facilitation, and inhibition effects). These findings suggest that as bilingual experience and cognitive skills increased, so did participants’ ability to attend to relevant stimulus information (i.e., arrow direction) and ignore irrelevant stimulus information (i.e., arrow location) on the Stroop arrows task. The patterns identified in these individual differences measures illustrate that bilingual experiences and cognitive performance jointly shape cognitive control.

Findings also suggest that linguistic factors can differ between the sociolinguistic contexts of the Midwest and SoCal bilinguals. In the current sample, participants from SoCal likely lived in a relatively more cooperative (integrated) language context in which both languages were used more regularly, while participants from the Midwest likely lived in a relatively more competitive (separated) linguistic environment. This language context distinction, along with individual differences variables, likely drove the sociolinguistic context effects in Stroop performance. The current work adds to a growing body of evidence that the language environments must be taken into consideration to understand how bilingual experience shapes cognitive control, and that the exact manner in which linguistic and cognitive variables shape cognitive control can vary across settings. The sociolinguistic context differences in the current study confirm our conclusion that not just bilingualism, but sociolinguistic environment, may shape cognitive control. The current study contributes to a call to examine the cognitive consequences of bilingualism in broader and more naturalistic contexts that take into account the sociolinguistic contexts of language use.

## Figures and Tables

**TABLE 1 |:** Participants’ language and cognitive background information.

	Midwest bilinguals mean (SD) *n* = 64	SoCal bilinguals mean (SD) *n* = 82	*P*-value
Age	23.27 (4.83)	22.72 (3.84)	0.12
Age of L2 (English) acquisition	5.95 (2.58)	5.12 (2.71)	0.06
Years of active bilingualism (age at testing-age of reported L2 fluency)	12.98 (4.88)	14.33 (5.33)	0.12
Current exposure to L2	64.94% (18.33)	55.11% (18.36)	0.001
Self-reported L1 proficiency (1–10 scale)	9.01 (1.01)	8.59 (1.24)	0.07
Self-reported L2 proficiency (1–10 scale)	9.12 (1.00)	9.13 (1.08)	0.96
L2 proficiency/exposure composite (z-score)	1.84 (1.54)	1.21 (1.38)	0.01
English receptive vocabulary (PPVT) standard score	109.13 (12.34)	100.50 (11.95)	<0.001
WASI matrix reasoning	28.33 (3.67)	26.54 (3.47)	0.01

**TABLE 2 |:** Linear mixed effects regression model for overall reaction times to congruent, incongruent, and neutral trials on the non-linguistic Stroop arrows task by sociolinguistic context.

Overall RT	B estimate	Std. error	Df	*t*-value	*p*-value
(Intercept) Congruent	0.9891	0.0004	202.10	2,751.961	<0.001
Incongruent	0.0280	0.0008	199.40	35.550	<0.001
Neutral	0.0084	0.0006	202.20	15.064	<0.001
Sociolinguistic context	−0.0009	0.0008	201.50	−1.209	0.023
Incongruent: sociolinguistic context	0.0048	0.0017	198.30	2.921	0.004
Neutral: sociolinguistic context	0.0061	0.0012	200.80	5.135	<0.001
Random effects estimates	Name	Variance	Std. dev.	Correlation	Correlation
Subject	(Intercept) Congruent	0.0000	0.004179		
	Incongruent	0.0001	0.009416	−0.89	
	Neutral	0.0000	0.005387	−0.96	0.81
Type	(Intercept)	0.0000	0		
Residual		0.0010	0.031164		

**TABLE 3 |:** Overall accuracy on the non-linguistic Stroop arrows task by trial type (congruent, incongruent, and neutral).

Trial type	Midwest bilinguals mean% (SD)	SoCal bilinguals mean% (SD)
Congruent	99.30 (1.67)	98.88 (1.50)
Incongruent	92.14 (9.40)	91.17 (7.39)
Neutral	98.88 (3.14)	98.51 (2.68)
Overall accuracy	96.78 (4.20)	96.18 (3.09)

**TABLE 4 |:** Summary of main effects of sociolinguistic context (A = Main effects of sociolinguistic context) and how they are modulated by individual differences measures (B = Individual differences across Spanish-English bilinguals).

	Stroop effect (incongruent–congruent)	Facilitation effect (neutral–congruent)	Inhibition effect (neutral-incongruent)
**A**			
Sociolinguistic context	**Midwest** < SoCal	**Midwest** < SoCal	Not significant
**B**			
Self-reported L2 proficiency and exposure	**Sociolinguistic context**+	**Sociolinguistic context+**↑Proficiency/Exposure, ↓Facilitation	
Age of L2 acquisition	**Sociolinguistic context+**	**Sociolinguistic context**+	
PPVT	**Sociolinguistic context**+	**Sociolinguistic context**+*↑PPVT,↓facilitation (led by Midwest)*	*↑PPVT, ↑inhibition (led by Midwest)*
WASI	**Sociolinguistic context**+(marginal) ↑WASI,↓Stroop *SoCal only: ↑WASI,↓Stroop*	**Sociolinguistic context**+*↑WASI,↓facilitation*	*SoCal only: ↑WASI,↓inhibition*

A. smaller effects = better performance. < less than, > greater than for categorical contrasts.

B. +main effect remains, −main effect disappears, ↑increased, ↓decreased for continuous contrasts.

Main effects of sociolinguistic context (in bold) and main effects/interactions with individual differences measures (in italics).

## Data Availability

The raw data supporting the conclusions of this article will be made available by the authors upon reasonable request.

## Footnotes

1 When script type was entered into the model as a covariate (1,200 ms response window; 1,500 ms response window, see Procedure), no main effect of script type or interaction between script type and trial types emerged (all ps > 0.1), confirming that script type did not influence performance.
